# Insulin-Receptor Substrate-2 (IRS-2) Is Required for Maintaining Glucokinase and Glucokinase Regulatory Protein Expression in Mouse Liver

**DOI:** 10.1371/journal.pone.0058797

**Published:** 2013-04-01

**Authors:** Isabel Roncero, Elvira Alvarez, Carlos Acosta, Carmen Sanz, Pedro Barrio, Veronica Hurtado-Carneiro, Deborah Burks, Enrique Blázquez

**Affiliations:** 1 The Center for Biomedical Research in Diabetes and Associated Metabolic Disorders (CIBERDEM), Madrid, Spain; 2 Departamento de Bioquímica y Biología Molecular, Facultad de Medicina, Universidad Complutense de Madrid-Instituto de Investigación Sanitaria del Hospital Clínico San Carlos (IdISSC), Madrid, Spain; 3 Centro de Investigación Príncipe Felipe, Valencia, Spain; 4 Departamento de Biología Celular, Facultad de Medicina, Universidad Complutense de Madrid, Madrid, Spain; University of Tübingen, Germany

## Abstract

Insulin receptor substrate (IRS) proteins play important roles in hepatic nutrient homeostasis. Since glucokinase (GK) and glucokinase regulatory protein (GKRP) function as key glucose sensors, we have investigated the expression of GK and GKRP in liver of *Irs-2* deficient mice and *Irs2(*−/−) mice where *Irs2* was reintroduced specifically into pancreatic β-cells [RIP-*Irs-2*/IRS-2(−/−)]. We observed that liver GK activity was significantly lower (p<0.0001) in *IRS-2*(−/−) mice. However, in RIP-*Irs-2*/IRS-2(−/−) mice, GK activity was similar to the values observed in wild-type animals. GK activity in hypothalamus was not altered in IRS-2(−/−) mice. GK and GKRP mRNA levels in liver of IRS-2(−/−) were significantly lower, whereas in RIP-*Irs-2*/IRS-2(−/−) mice, both GK and GKRP mRNAs levels were comparable to wild-type animals. At the protein level, the liver content of GK was reduced in IRS-2(−/−) mice as compared with controls, although GKRP levels were similar between these experimental models. Both GK and GKRP levels were lower in RIP-*Irs-2*/IRS-2(−/−) mice. These results suggest that IRS-2 signalling is important for maintaining the activity of liver GK. Moreover, the differences between liver and brain GK may be explained by the fact that expression of hepatic, but not brain, GK is controlled by insulin. GK activity was restored by the β-cell compensation in the RIP-*Irs-2*/IRS-2 mice. Interestingly, GK and GKRP protein expression remained low in RIP-*Irs-2*/IRS-2(−/−) mice, perhaps reflecting different mRNA half-lives or alterations in the process of translation and post-translational regulation.

## Introduction

Insulin receptor substrate-2 (IRS-2) integrates insulin and IGF-I receptor signals that are transmitted through transduction pathways to produce growth and metabolic effects. IRS-2 is especially important in hepatic nutrient homeostasis as it mediates the anabolic effects of insulin through the PI3K-Akt cascade [1], [2] and suppresses gluconeogenesis and apoptosis [3], [4]. Mice lacking IRS-2 develop diabetes due to peripheral insulin resistance, failed hypothalamic regulation of appetite and β-cell insufficiency [5]. These mice have additional phenotypes, including a 40% reduction in brain mass [6].

The importance of IRS-2 signals in β-cell development and function is supported by several lines of research. When *Irs-2* was reintroduced specifically in pancreatic β-cells using the rat insulin II promoter (RIP), *Irs-2*-deficient mice did not develop diabetes, as the expression of this signalling molecule restored β-cell compensation [7]. The loss of *Irs-2* expression in β-cells has been linked to type 2 diabetes in humans: microarray comparison revealed that *Irs-2* is significantly reduced in islets of patients with type 2 diabetes, as compared with controls [8].

Glucokinase (GK) is the type IV isoform of the hexokinase family [9] that catalyses the formation of glucose-6-phosphate. It has a low affinity for glucose and is not subjected to feedback inhibition by glucose-6-phosphate, whereby it works as a pacemaker in glucose oxidation through glycolysis. GK is expressed mainly in the liver [9], pancreatic β-cells [10] and the brain [11], [12], [13], where it acts as a glucose sensor. This enzyme is encoded by a single gene, but the presence of alternative promoters allows the cell-specific expression of this protein with differential regulation. Thus, liver GK is regulated by insulin [14], whereas in the endocrine pancreas and the brain [15], [16] it seems to be controlled by glucose levels.

GK is considered the primary glucose sensor in pancreatic β-cells and is involved in glucose-dependent insulin release [17]. Mutations of GK produce MODY-type monogenic diabetes [18]. We have posited [12], [19] that hypothalamus GK functions as a glucose sensor involved in the control of feeding behaviour, and that GLP-1, an anorexigenic peptide, may modulate this function by altering glucose metabolism in human hypothalamus [20].

Given the control of glucose metabolism by GK in the liver and its role as a glucose sensor, this enzyme has been considered a target for the development of anti-diabetic agents [21]. These molecules, called glucokinase activators (GKA) stimulate glycolysis and glycogen synthesis [22] in isolated rat hepatocytes. Furthermore, these compounds decrease glucose circulating levels and stimulate insulin secretion in experimental animals and in type 2 diabetes patients [23], [24]. GK not only functions as a glucose sensor in insulin secretion by pancreatic β-cells, but in addition it is now known that this enzyme is involved in the regulation of β-cell mass. Accordingly, a link between GK and IRS-2 is required for compensatory β-cell hyperplasia in response to insulin resistance induced by a high-fat diet [5].

Binding the glucokinase regulatory protein (GKRP) to GK blocks the activity of the enzyme, with this effect being controlled by fructose esters [25], [26]. Thus, fructose-6-phosphate reinforces, and fructose-1-phosphate antagonises, the association of the enzyme-inhibitor complex. In the basal state, both GK and GKRP are bound in the nucleus, but in the postprandial state, glucose and fructose circulating levels rise, and then GK is released from GKRP and remains free in the cytoplasm ready to phosphorylate glucose. GKRP functions as an allosteric inhibitor of GK and as a metabolic sensor that binds and transports GK to the nucleus. Both proteins may then participate in glucose sensing and metabolic regulation.

Considering that IRS-2 plays an important role in hepatic nutrient homeostasis, we have investigated the expression of GK and GK activity and also GKRP expression in the liver, using two experimental models of insulin receptor substrate-2 deficiency in mice: IRS-2 deficient mice that develop type 2 diabetes at 10–12 weeks of age and IRS-2 deficient mice in which *Irs-2* was specifically reintroduced into pancreatic β-cells [RIP-*Irs-2*/IRS-2(−/−)] to restore pancreatic β-cell compensation. Significant alterations of liver GK and GKRP were found in both experimental approaches.

## Materials and Methods

### Ethics Statement

This study was carried out in strict compliance with the recommendations of the European Union Guidelines for the Care and the Use of Laboratory Animals (European Economic Community Directive 86/609/EEC) and all the experimental protocols were approved by the Institutional Animal Care and Use Committee of Madrid Complutense University. All procedures were performed under isoflurane anesthesia, and all efforts were made to minimize suffering.

### Animals

*Irs-2*-deficient mice were generated initially on a C57BL6/J: SV129 background [27] and then backcrossed to establish a pure C57BL6/J background [7]. Thus, the offspring resulting from the breeding of *Irs-2*(−/−) with RIP-*Irs-2* line were C57BL6/J. The generation and genotyping of the *Irs-2*−/− and the RIP-*Irs-2*(−/−) models have been described previously [7], [27]. Animals were housed under standard conditions with a 12 h/12 h light/dark cycle (light on at 8:00 am) with ad libitum access to food and water. Prior to sacrifice for collection of tissues, mice were fasted overnight and then were sacrificed by cervical dislocation. Females IRS-2(−/−) and their control wild type of 6-7 week old as well as and RIP-*Irs2*-IRS-2(−/−) and their control wild type of ∼16 months old were used in our studies. Although the RIP-*Irs2*-IRS-2(−/−) transgene restores perfectly β-cell compensation in male *Irs-2*(−/−) mice [28], we chose to use females in our study in an effort to minimize the impact of failed β-cell compensation on hepatic function in the group of young animals. Most *Irs-2* deficient males develop severe hyperglycemia between 8–12 weeks [29], whereas the onset of diabetes in females occurs later (12–16 weeks) and less severely [30].

### Blood plasma glucose and insulin levels determinations

Glucose levels were sampled from mouse tail bleeds using a Glucometer Elite (Bayer Corp., Elkhart, Indiana, USA). Blood plasma insulin levels were determined using a competitive ELISA KIT (Millipore, MA, USA), following the manufacturer's instructions.

### RNA isolation

Total liver RNA was isolated with TRIZOL (Life Technologies, Barcelona, Spain).

### Real-time polymerase chain reaction

RT was carried out with 1 μg of RNA. For real-time PCR, a TaqMan assay was used (Applied Biosystems). Primers and probes were designed with Primer Express 2.0 software from Applied Biosystems (*Gck, Gkrp* and *18S RNA*). Each reaction contained 4 μl of a 1∶10 dilution of template cDNA at a final volume of 20 μl. The housekeeping *18S RNA* gene was used, adding 4 μl of a 1∶1000 dilution of template cDNA. All samples were run in duplicate on a 7300 Sequence Detection System (Applied Biosystems).

### Detection of GK and GKRP proteins by Western blotting

Tissue was homogenised in RIPA buffer (150 mM NaCl, 50 mM Tris·HCl, pH 7.2, 1% sodium deoxycholate, 1% Triton X-100, 0.25 mM EDTA, pH 8.0, 10 mM NaF, and 1 mM sodium orthovanadate) and a protease inhibitor cocktail tablet (Roche Diagnostics, Mannheim, Germany). 30 μg of total protein was separated by 10% SDS-PAGE and transferred onto a polyvinylidene fluoride membrane (Millipore, MA, USA) [13].

For immunoblot detection of GK and GKRP, after blocking for 1 h in phosphate-buffered saline (PBS)-0.1% Tween-20 containing 5% bovine serum albumin, the membranes were incubated either with a rabbit polyclonal antiserum against GK (anti-GKC H88), or with a goat polyclonal antiserum against GKRP (anti-GCKR N-19), diluted 1∶1000, with both provided by Santa Cruz Biotechnology (Santa Cruz, CA, USA). After washing off the unbound antibodies, the membranes were incubated with anti-rabbit or anti-goat immunoglobulin Gs conjugated to horseradish peroxidase (1∶5000) for 1 h at room temperature. Chemiluminescence detection was carried out in the presence of Chemiluminescent Horseradish Peroxidase Substrate (Millipore, MA, USA).

Membranes were also probed with anti-β-actin antibody (Sigma-Aldrich, Inc., St Louis, MO, USA) to standardise protein loading for the different samples. The resulting bands were quantified in a Bio-Rad GS-800 Calibrated Densitometer (Bio-Rad, Hercules, CA, USA) using Quantity One software.

### Assay of GK-phosphorylating activities

Liver tissue were homogenised in an ice-cold lysis medium (50 mM HEPES, 150 mM NaCl, 5 mM MgCl_2_, and 1 mM EDTA, pH 7.4) supplemented with 1 mM DTT, 1 mM PMSF and 10 μM leupeptine. The homogenate was used to measure enzyme activities [13].

Glucose-phosphorylation was measured using a spectrophotometric assay [13]. The activity analysis involved assays at two glucose concentrations: 0.3 mM for low-Km hexokinase (HK) activities, a concentration at which GK is essentially inactive and 30 mM glucose, a concentration at which all phosphotransferase activities were measured. GK activity was calculated by subtracting the glucose-phosphorylating activity at 0.3 mM glucose from the total activity measured at 30 mM glucose.

### Statistical Analyses

All values are presented as means ± SEM. Comparisons among groups were made using ANOVA. P<0.05 was considered statistically significant.

## Results

### Characterization of GK activity in liver and hypothalamus of *Irs-2*(−/−) and RIP-*Irs-2*/IRS-2(−/−) mice

**Figure 1:**
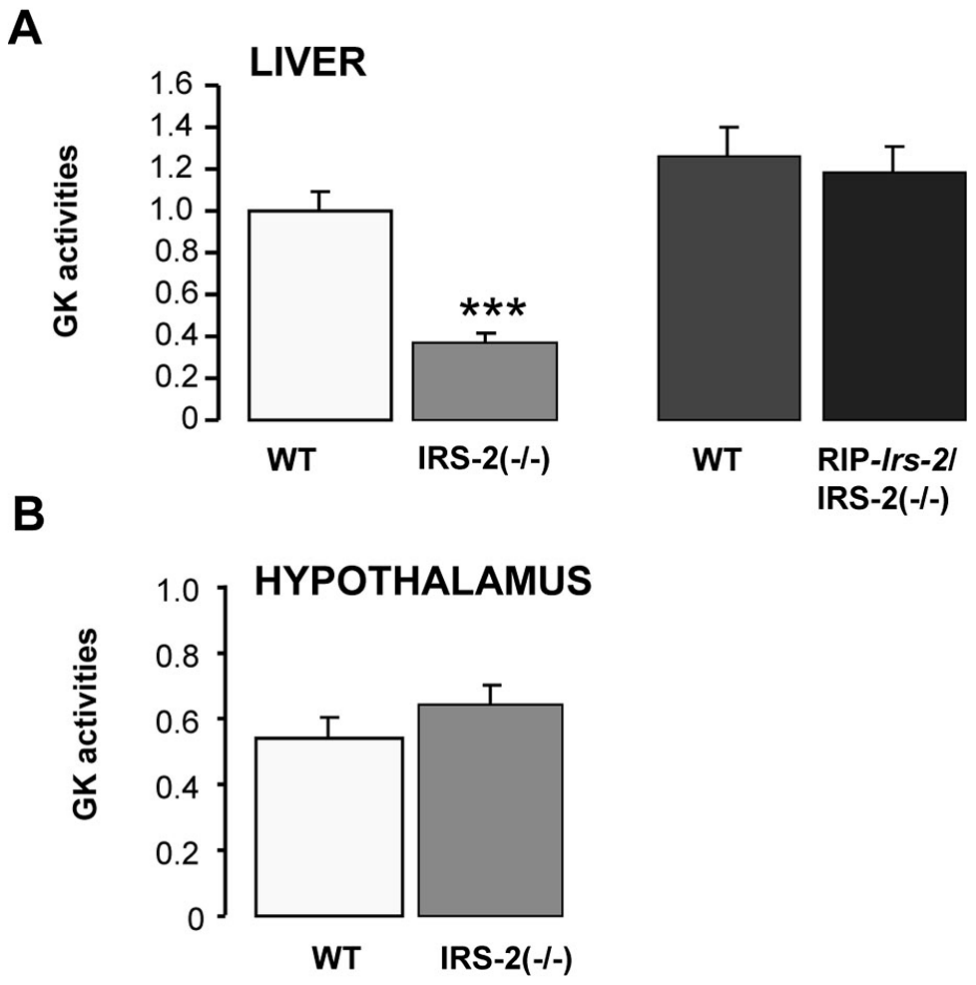
Glucokinase activity in soluble fractions of liver and hypothalamus. Extracts were prepared from liver and hypothalamus of female mice of the indicated genotypes. GK activity was calculated by subtracting the glucose-phosphorylating activity at 0.3 mM glucose from the total activity measured at 30 mM glucose. The bars represent means ± SE. n = 8 IRS-2(−/−) (6–7 weeks old), n = 10 WT (6–7 weeks old), n = 5 RIP-*Irs-*2/IRS-2(−/−) at ∼16 months of age, and n = 5 WT (at ∼16 months of age). GK activity in liver from wild type mice (6–7 weeks old) was considered as 1. *** p<0.001 (IRS-2(−/−) *vs* WT mice).

Consistent with published results [27], both blood plasma glucose and insulin concentrations were higher (p<0.05) in IRS-2(−/−) mice as compared with their wild type controls. Blood plasma glucose was similar between RIP-*Irs-2*/IRS-2(−/−) mice and their wild type controls, but insulin circulating levels were significantly greater in RIP-*Irs-2*/IRS-2(−/−) (p<0.05) than their wild type controls (Table 1). Liver glucokinase activity decreased significantly (p<0.001) in IRS-2(−/−) mice as compared with their wild-type controls (Fig. 1A). However, when IRS-2 was specifically reintroduced into pancreatic β-cells in IRS-2(−/−) mice, liver GK activity was restored to levels of control mice (Fig. 1A). There was no difference in the values of GK activity between control mice of both ages. In the hypothalamus, GK activity was similar between genotypes (Fig. 1B). GK activity in the hypothalamus was significantly lower than in the liver. As previously reported [6], brain weight was decreased significantly (p<0.05) in IRS-2(−/−) mice and the reduced brain weight was also noted in RIP-*Irs-2*/IRS-2(−/−), as compared with the wild type animals (Figure S1).

**Table 1:** Glucose and insulin values in peripheral blood.

Mice	Glucose (mg/dl)	Insulin (ng/ml)
WT	91.2±3.9 (9)	0.37±0.01 (4)
IRS-2(−/−)	135.7±8.2 (9)*	1.38±0.06 (4)*
WT	83.2±1.6 (5)	4.00±0.32 (5)
RIP-IRS-2(−/−)	92.9±1.9 (5)	6.85±0.38 (5)#

The values are given as the mean ± SEM of the number of experiments in parentheses. * p<0.05 WT *vs* IRS-2(−/−); # p<0.05 WT vs RIP-IRS-2(−/−).

### GK and GKRP mRNA levels are reduced in IRS-2(−/−) liver

**Figure 2:**
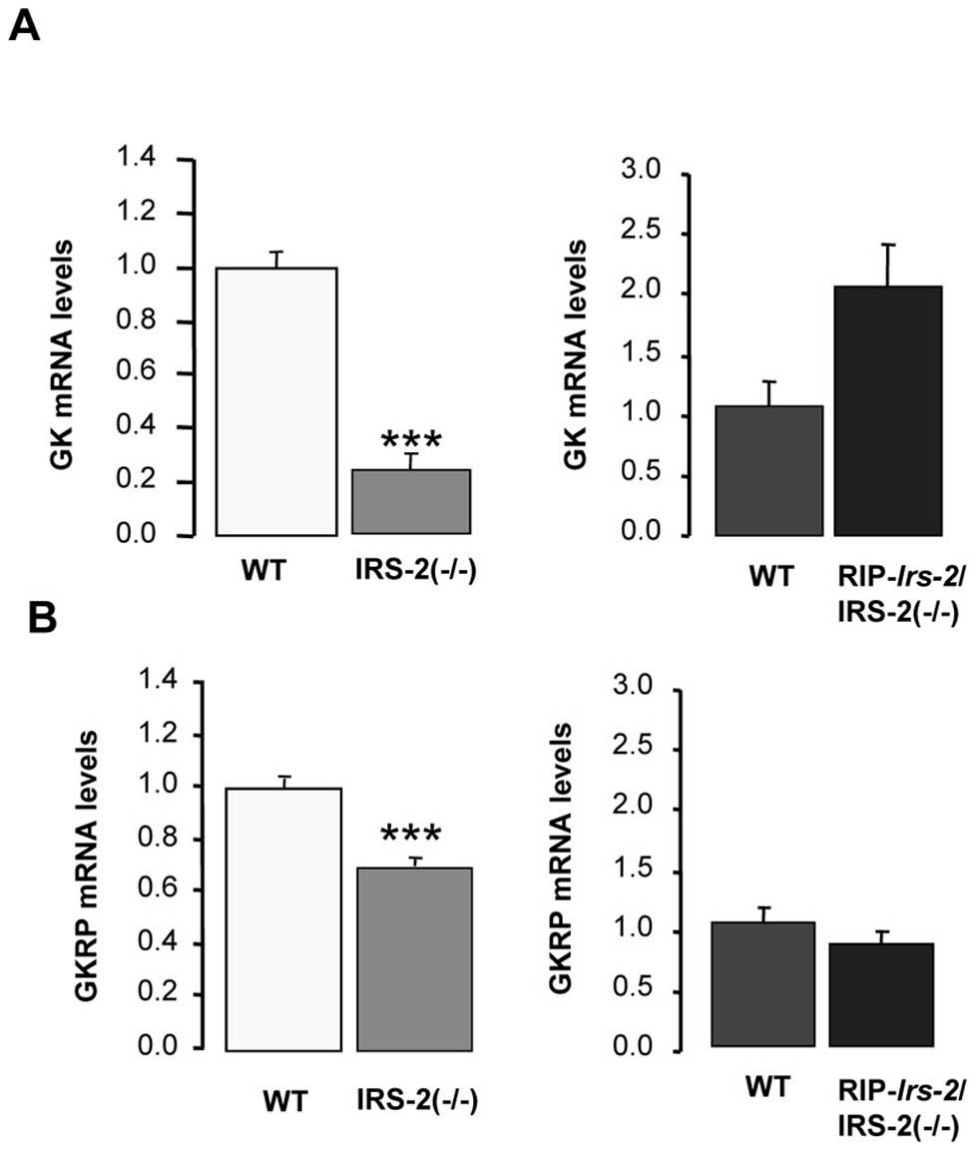
GK and GKRP mRNA levels in livers from IRS-2(−/−), RIP-*Irs-*2/IRS-2(−/−) and their wild type mice. Levels of mRNA encoding GK (**A**) and GKRP **(B**) were measured in liver from IRS-2(−/−) or from RIP-*Irs-2*/IRS-2(−/−) and their wild-type mice. The bars represent means ± SE (n = 8 animals per experimental group). The values obtained in wild type mice were considered as 1. *** p<0.001 (IRS-2(−/−) *vs* WT mice).

To determine whether the reduced GK activity in liver were due to altered GK gene expression, the mRNA levels of GK and GKRP were measured by RT-PCR. A significant reduction (p<0.001) in both genes was observed in the IRS-2(−/−) mice (Fig. 2), suggesting that the reduced GK activity was related to a decreased gene expression. Interestingly, GK and GKRP mRNAs levels in the RIP-*Irs-2*/IRS-2(−/−) (Fig. 2) were comparable to age-matched controls, suggesting that beta cell compensation in this model is sufficient to restore GK mRNA and enzymatic activity.

### Liver GK and GKRP protein contents in IRS-2(−/−) and in RIP-*Irs-2*/IRS-2(−/−) mice

**Figure 3:**
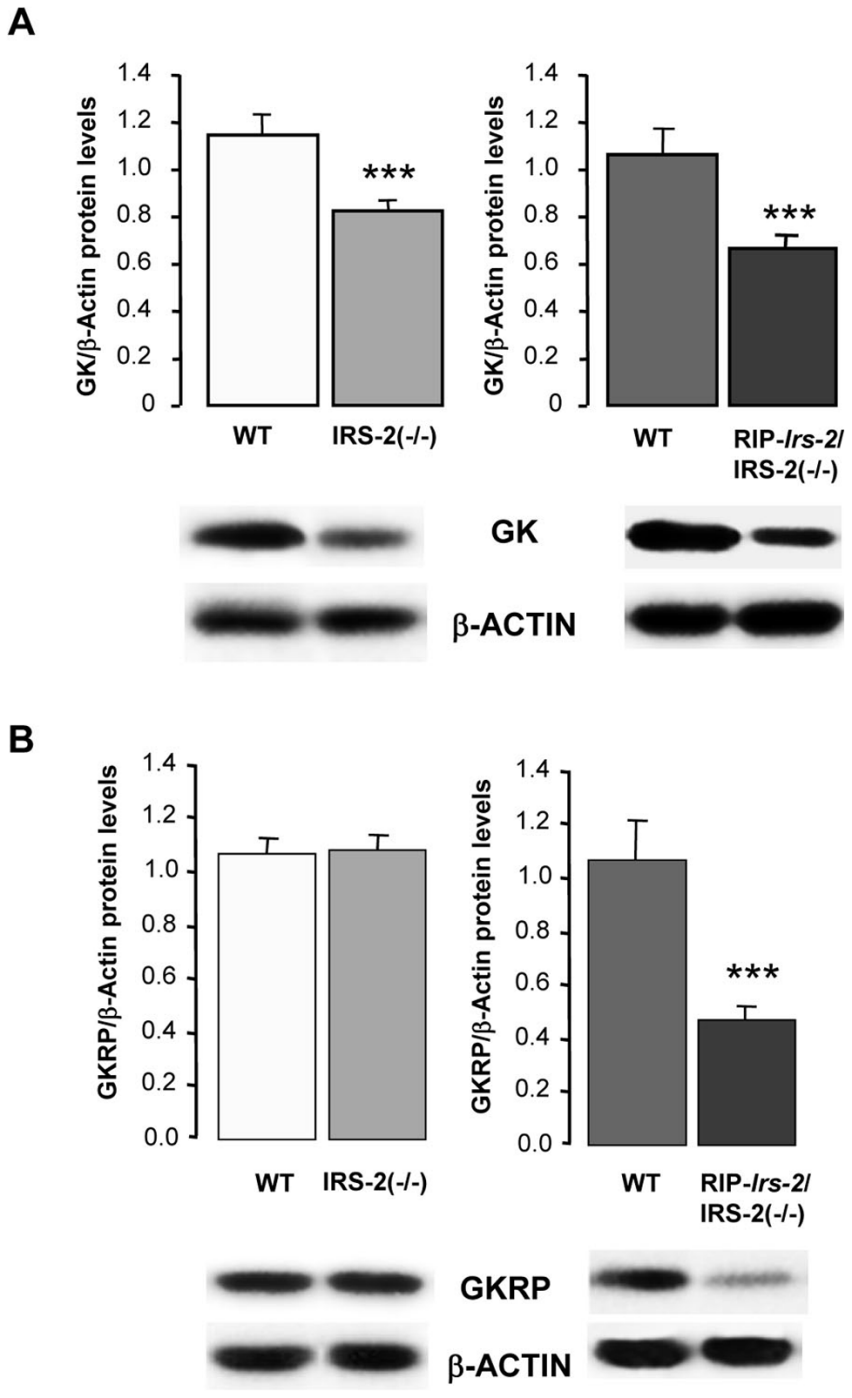
Analysis of GK and GKRP protein expression in liver from IRS-2(−/−), RIP-*Irs-*2/IRS-2(−/−) and their wild type mice. Levels of total GK (**A**) and GKRP (**B**) were assessed by Western blots of liver lysates prepared from IRS-2(−/−) or from RIP-*Irs-2*/IRS-2(−/−) and their wild type mice. The bars represent means ± SE of the densitometric values normalized by β-actin (n = 5–8 animals per group). The values obtained in wild type mice were considered as 1. *** p<0.001 (IRS-2(−/−) or RIP-*Irs-*2/IRS-2(−/−) *vs* their WT mice). Representative Western blots images are shown.

**Figure 4:**
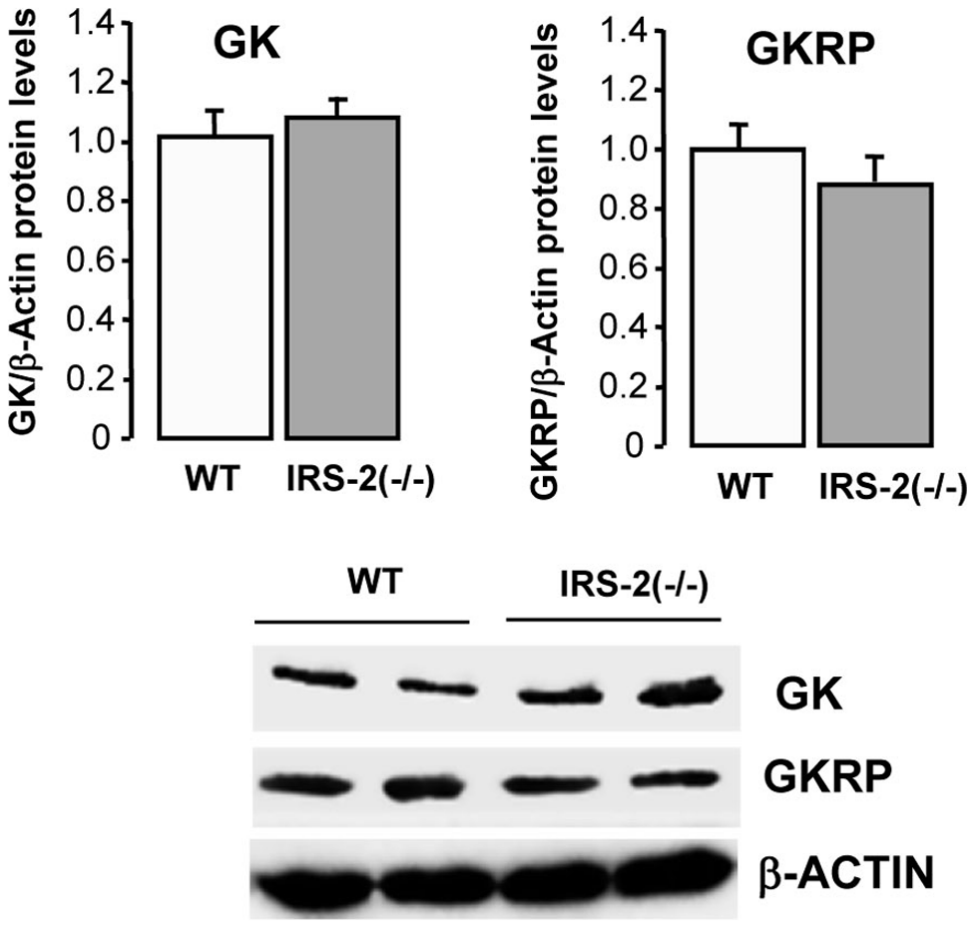
Analysis of hypothalamic GK and GKRP protein expression in IRS-2(−/−) and wild type mice. Total protein levels of GK and GKRP in hypothalamus from IRS-2(−/−) and their wild type mice. The bars represent means ± SE of the densitometric values normalized by β-actin (n = 3 animals per group). The values obtained in wild type mice were considered as 1. Representative Western blots images are shown.

Hepatic GK protein expression was determined by Western blotting and was significantly lower (p<0.05) in IRS-2(−/−) mice as compared with their wild-type controls (Fig. 3A), consistent with the lower level of GK activity in this model. However, in the RIP-*Irs-2*/IRS-2(−/−), GK protein was lower than control animals, contrasting the normal GK mRNA levels and activity observed in this model. There was no difference in the values of GK activity between control mice of both ages. Expression of liver GKRP protein was similar in IRS-2(−/−) mice as compared with their wild-type controls, whereas it was decreased in the RIP-*Irs-2*/IRS-2(−/−) (Fig. 3B). We also examined hypothalamic GK and GKRP protein expression but observed no significant differences between the wild-type and IRS-2 deficient mice, consistent with the analysis of GK activity (Fig. 4).

## Discussion

Body fuel is acquired in a discontinuous manner through the ingestion of food and therefore, mechanisms exist to maintain a constant supply of nutrients for peripheral tissues and particularly for the brain which has an exquisite requirement for glucose. The liver is responsible for the storage and distribution of endogenous nutrients, as controlled by the metabolic hormones insulin and glucagon. Both GK and GKRP serve as glucose sensors and modulate the metabolic fate of nutrients in liver. GK functions as a pacemaker in glucose oxidation through glycolysis, while at the same time, it metabolises large amounts of glucose from the gut after food intake. This effect is increased because liver GK is regulated by insulin [14], while pancreatic and brain GK seem to be controlled post-translationally by glucose levels [15], [16]. On the other hand, the kinetic properties of the low Km hexokinases indicate they are saturated at 1 mM glucose, after which they are unable to detect changes at physiological glucose concentrations. This may be the reason that GK is the more abundant hexokinase in liver, as this organ has to metabolise, store and distribute large amounts of glucose.

The findings of the present study provide new insights regarding the role of IRS-2 in regulating hepatic GK. Deletion of *Irs-2* in mice is known to generate insulin resistance which is particularly pronounced in liver [27]. Our data reveal that *Irs-2*-deficiency in mice has profound effects on GK and GKRP expression and activity in the liver but not the hypothalamus. Interestingly, the impairment of GK activity was rescued in the RIP-*Irs-2*/IRS-2(−/−) model where restored IRS-2 function in pancreatic beta cells provides long-term compensation for peripheral insulin resistance. Consistent with this notion, the insulin levels in the double transgenic mice were significantly higher than age-matched control mice and also more elevated than in simple IRS-2(−/−) mice. Activation of insulin receptor is required for triggering two major metabolic actions of insulin, namely, the activation of glycogen synthesis and the inhibition of gluconeogenic enzymes gene expression [1], [2], [3], [4]. These metabolic effects require IRS-2 signalling through AKT/GSK-3 and an atypical protein kinase Cζ/λ [3]. The activation of this pathway is also necessary for regulating GK gene expression [31], [32]. Here we present experimental evidence to indicate that IRS-2 is required to maintain normal levels of GK and GKRP gene expression and GK activity in the liver. Both GK and IRS-2 are also required for β-cell compensation in response to high-fat diet [5].

Thus, although insulin levels were elevated in fasted conditions in IRS-2(−/−) mice, expression of mRNAs coding for GK and GKRP was reduced, since in this model moderate hyperinsulinemia cannot compensate for the presence of severe hepatic insulin resistance. Consistent with this, protein levels and GK activity were also lower in IRS-2-deficient mice compared to control mice. In contrast, hypothalamic glucokinase activity and protein expression were similar to wild type animals. These data are in agreement with the presence of tissue-specific promoters in the GK gene that modulate differential regulation [33]. The upstream promoter, classified as the neuroendocrine promoter to distinguish it from the hepatic promoter, is functional in pancreatic β-cells and in the brain, while the downstream promoter is used only in the liver. GK activity in β-cells and in the brain appears to be controlled by glucose. In contrast, the liver-specific promoter is affected mainly by insulin and glucagon, which explains the extraordinary transcriptional regulation by the nutritional state.

Data from other laboratories have demonstrated that GK expression was reduced upon loss of *Irs1* expression in liver using an adenovirus-mediated RNA interference technique, whereas knockdown of *Irs-2* led to increased levels of the transcription factor SREBF1c [34]. In addition to regulating the expression of lipogenic enzymes, SREBF1c has been shown to mediate GK induction by insulin [35]. In our study, the level of hepatic triglycerides was comparable between IRS-2 deficient mice and control mice (data not shown). Similar results have been reported in mice with liver-specific deletion of IRS-2 [36].

Recently, it has been proposed that IRS-2 has a key role during fasting. *Irs-2* expression is high in the fasted state and significantly reduced by feeding and by increased insulin levels [37]. Insulin resistance in liver contributes greatly to the development of type 2 diabetes mellitus [38]. Indeed, liver-specific knockout of the insulin receptor has demonstrated the critical role of hepatic insulin resistance in metabolic abnormalities [39]. Furthermore, IRS-2(−/−) mice have confirmed that insulin signalling defects in the liver, but not in skeletal muscle or adipose tissue, play a major role in the development of diabetes, particularly in combination with pancreatic β cell dysfunction [5], [27]. Reduced expression of IRS-2 has been described in a number of “in vivo” models of insulin resistance [40], [41]. Both IRS-1 and IRS-2 have complementary roles in the control of hepatic metabolism [34], as knock-down studies have shown that either or both can regulate PI3K activity and hepatic metabolism. Using mice with altered expression of hepatic IRS-1 and IRS-2, Guo et al. [36] also reported that IRS-1 is the major intermediary of the insulin signalling to maintain glucose homeostasis in the liver. In addition, IRS-1 or IRS-2 function to modulate hepatic gene expression needed for control glucose homeostasis and maintaining normal hepatic function and growth [42].

Interestingly, our data suggest that liver GK activity can be rescued in IRS-2 deficient mice by maintaining pancreatic β-cell compensation via expression of the RIP-*Irs2* transgene; GK activity and mRNAs expression of GK and GKRP in RIP-*Irs-2*/IRS-2(−/−) mice were comparable to wild type mice. Restoration of *Irs2* specifically to β-cells has been demonstrated to prevent the development of diabetes caused by IRS-2 deficiency and diet-induced obesity [7]. Restoration of GK expression in liver was reported to normalize glucose levels in Zucker diabetic fatty rats [43]. Surprisingly, reduced expression of GK protein was also observed in RIP-*Irs-2*/IRS-2(−/−) mice. This apparent paradox could be due to differences in the process of translation, mRNA stability, or to changes in the mechanisms of post-translational regulation. GK activity and expression may be regulated by GKRP. Thus, overexpression of GKRP increases GK activity and expression, whereas in GKRP knockout mice, GK protein expression and activity are reduced due to posttranscriptional regulation of GK [44], [45]. In our study, protein levels of GKRP were not modified in IRS-2(−/−) deficient mice, although the mRNA coding to GKRP was lower in RIP-*Irs-*2/IRS-2(−/−) mice. Our data suggest that both hepatic proteins may be regulated at transcriptional and posttranscriptional level and insulin signalling can influence both processes.

In conclusion, our results implicate a role for IRS-2 in regulating liver GK gene expression and catalytic activity, which ensure active glucose metabolism in this organ. Our findings in IRS-2 deficient mice suggest that IRS-2 is required for nutrient homeostasis in hepatocytes where GK is induced by insulin. In addition, GKRP also requires IRS-2 signals to potentiate GK activity. The reduced GK activity in IRS-2(−/−) mice may be due to insulin resistance in these animals since this impairment was overcome by introduction of the RIP-*Irs-2* transgene to restore β-cell compensation.

## Supporting Information

Figure S1 Brain weight of IRS-2(−/−), RIP-*Irs-*2/IRS-2(−/−) and their wild type mice. The bars represent means ± SE of the brain weight (n = 5–8 animals per group). * p<0.05 (IRS-2(−/−) or RIP-*Irs-*2/IRS-2(−/−) *vs* their WT mice).
(JPG)
