# Supplementary figures and images for: Insulin-Receptor Substrate-2 (IRS-2) Is Required for Maintaining Glucokinase and Glucokinase Regulatory Protein Expression in Mouse Liver
Source: PLoS One. 2013 Apr 1;8(4):e58797. doi: 10.1371/journal.pone.0058797 (PMC3613347; doi:10.1371/journal.pone.0058797)

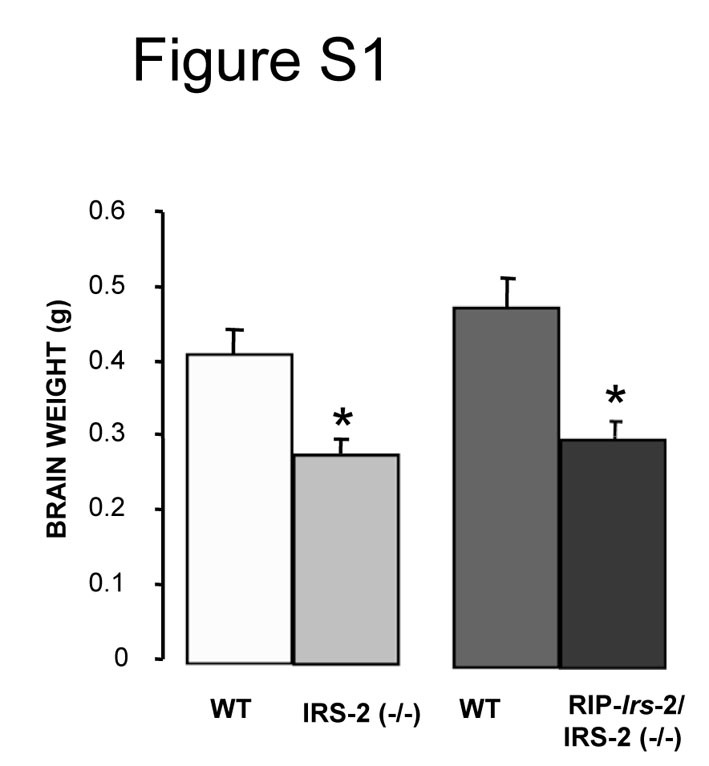

Supplement: Figure S1 — Brain weight of IRS-2(−/−), RIP-Irs-2/IRS-2(−/−) and their wild type mice. The bars represent means ± SE of the brain weight (n = 5–8 animals per group). * p<0.05 (IRS-2(−/−) or RIP-Irs-2/IRS-2(−/−) vs their WT mice). (JPG) [file pone.0058797.s001.jpg]
